# COVID-19 Community Incidence and Associated Neighborhood-Level Characteristics in Houston, Texas, USA

**DOI:** 10.3390/ijerph18041495

**Published:** 2021-02-04

**Authors:** Abiodun O. Oluyomi, Sarah M. Gunter, Lauren M. Leining, Kristy O. Murray, Chris Amos

**Affiliations:** 1 Section of Epidemiology and Population Sciences, Department of Medicine, Baylor College of Medicine, Houston, TX 77030, USA; chris.amos@bcm.edu; 2 Environmental Health Service, Department of Family and Community Medicine, Baylor College of Medicine, Houston, TX 77030, USA; 3 Gulf Coast Center for Precision Environmental Health, Baylor College of Medicine, Houston, TX 77030, USA; 4 National School of Tropical Medicine, Baylor College of Medicine, Houston, TX 77030, USA; sm22@bcm.edu (S.M.G.); Lauren.Leining@bcm.edu (L.M.L.); kmurray@bcm.edu (K.O.M.); 5 Department of Pediatrics, Texas Children’s Hospital, Baylor College of Medicine, Houston, TX 77030, USA; 6 William T. Shearer Center for Human Immunobiology, Texas Children’s Hospital, Houston, TX 77030, USA; 7 Division of Epidemiology, Human Genetics, and Environmental Sciences, School of Public Health, The University of Texas Health Science Center at Houston, Houston, TX 77030, USA; 8 Institute for Clinical and Translational Research, Baylor College of Medicine, Houston, TX 77030, USA

**Keywords:** COVID-19, neighborhood inequity, geographic information system, social determinants of health, spatial epidemiology, geographically weighted regression

## Abstract

Central to developing effective control measures for the COVID-19 pandemic is understanding the epidemiology of transmission in the community. Geospatial analysis of neighborhood-level data could provide insight into drivers of infection. In the current analysis of Harris County, Texas, we used custom interpolation tools in GIS to disaggregate COVID-19 incidence estimates from the zip code to census tract estimates—a better representation of neighborhood-level estimates. We assessed the associations between 29 neighborhood-level characteristics and COVID-19 incidence using a series of aspatial and spatial models. The variables that maintained significant and positive associations with COVID-19 incidence in our final aspatial model and later represented in a geographically weighted regression model were the percentage of the Black/African American population, percentage of the foreign-born population, area derivation index (ADI), percentage of households with no vehicle, and percentage of people over 65 years old inside each census tract. Conversely, we observed negative and significant association with the percentage employed in education. Notably, the spatial models indicated that the impact of ADI was homogeneous across the study area, but other risk factors varied by neighborhood. The current findings could enhance decision making by local public health officials in responding to the COVID-19 pandemic. By understanding factors that drive community transmission, we can better target disease control measures.

## 1. Introduction

The novel coronavirus disease (COVID-19; caused by SARS-CoV-2) was declared a global health emergency by the World Health Organization on 30 January 2020 [1]. By mid-November 2020, there were more than 60 million cases worldwide, with over 13 million cases occurring in the United States (US) alone, according to the Johns Hopkins University COVID-19 dashboard [2,3]. The current outbreak of COVID-19 has led to an unprecedented impact on daily life and exposed critical weaknesses in the public health infrastructure in the US. Controlling COVID-19 requires swift identification and containment of cases and contacts to prevent further community transmission [4]. Understanding the transmission dynamics within community settings and determining which groups are at the highest risk of infection is the cornerstone of public health interventions for reducing COVID-19 morbidity and mortality. Geospatial analytics could represent an important tool for determining community level risk factors, including social determinants of health (SDOH).

Examining neighborhood-level stressors and assets provides an important framework for understanding the SDOH [5]. Simultaneously, at least in the US, renewed attention is directed toward the significance of the SDOH in contemporary health care discourse [6,7,8]. Important aspects of the SDOH include poverty, low educational attainment, rapid urbanization and substandard housing, and lack of employment opportunities [9,10]. Additional SDOH relevant to COVID-19 include occupational risks from essential work, multigenerational households, homelessness, and food insecurity [11]. Given the fact that neighborhood-level social, economic, and environmental factors have both direct and indirect effects on health [12,13,14], understanding how they affect the community spread of COVID-19 is invaluable. In essence, knowledge gained about the spatial structures of any relationship between SDOH and COVID-19 may be used to plan and target intervention programming differently across a given study area [15,16].

Across the US, researchers have identified spatiotemporal trends in COVID-19 incidence, determined case-fatality rates [17,18], and compared the spatial patterns of socioeconomic variables to identify the factors that correlate with mortality in urban and rural settings [19]. Studies have reported that significant neighborhood-level inequities underlie the variance in COVID-19 community incidence and mortality rates in the USA [20,21,22]. To date, studies that have conducted geospatial analysis of COVID-19 within communities have predominantly used county [17,19,23,24,25,26,27,28] or zip code [29] as their primary unit of analysis. Meanwhile, the geographic unit used in any area-based analysis is fundamentally important for how precise estimates of reality are, while also enhancing the generalizability of findings and reducing bias. Quantifying the associations between COVID-19 and relevant outcomes aggregated to the county and zip code levels may obscure the heterogeneity of both the dependent and independent outcomes of interest [29]. In general, smaller geographic units provide more accurate estimates of neighborhood-level characteristics, the only exception being situations where the number of records available at the smaller geography is too small to represent stable estimates of the outcome of interest [30,31]. Therefore, census tract may represent an ideal unit of analysis for neighborhood-level characteristics. Additionally, several socioeconomic and demographic data are available at the census tract level. Providing COVID-19 surveillance data at census tract levels may facilitate spatial analysis of related phenomena and potentially benefit public health response efforts. We believe our current analysis using census-tract level data overcomes the limitations faced by other analysis using larger units of measure. We are unaware of any current reporting or surveillance systems in the USA that provide COVID-19 data below the zip code level while integrating SDOH.

In the current analysis, we used extended spatial analytic procedures to disaggregate COVID-19 community incidence estimates provided at the zip-code geographic unit into census tract estimates. Subsequently, we assessed the associations between census tract measures of SDOH and the community incidence of COVID-19 using a series of aspatial and spatially weighted regression models to determine neighborhood drivers of disease transmission in Harris County, Texas, the most diverse county in the USA and the third most populous with 4.7 million people [32].

## 2. Materials and Methods

### 2.1. Study Setting

Harris County, which includes the City of Houston, is home to a racially and ethnically diverse population, having more than double the USA proportion of foreign-born residents. Houston’s population can be characterized as 44.8% Hispanic, 24.6% non-Hispanic white, 22.5% non-Hispanic African American, and 6.9% Asian [33]. Houston also has a large underserved population with one of the highest uninsured rates in the nation at more than double the national average (25.4% versus 10%) and a high poverty level (20.6% for Houston vs. 11.8% nationally) [33]. Finally, residents in Harris County have the highest diversity in life expectancy of any US county, ranging from over 85 years of age to 65 years of age in regions that are less than 5 miles apart [34].

For this study, we used the USA Census Bureau’s census tract geography (i.e., neighborhoods) as the unit of analysis for the current study. All the census tracts in Harris County (*N* = 786) were considered for inclusion in our analysis. The census tract is a small and relatively permanent statistical subdivision of a county that is designed to be homogeneous in terms of population characteristics, economic status, and living conditions. Nationally, census tracts typically have between 1000 and 8000 inhabitants and vary in land size, with an optimum population of 4000 residents or 1600 housing units [35].

### 2.2. Dependent Variable

#### COVID-19 Neighborhood-Level Community Incidence

Around the third week in April 2020, the City of Houston and Harris County jointly created an online dashboard that reports the total number of COVID-19 cases diagnosed among Harris County residents. Cases were aggregated to the zip code level and displayed on a web map [36]. For this analysis, we included only incident cases reported between 23 June 2020 and 3 August 2020. Our study period focuses on a spike in COVID-19 incidence and mortality across Texas following the start of Phase III opening of businesses across the state, which started in early June 2020. Previously, on 30 March 2020, all non-essential businesses were required to close due to the pandemic and had gone through a staged reopening (phase I on May 1 and phase II on May 18). Phase III allowed all businesses to operate at up to 50% capacity. Additionally, some businesses were able to operate at 100% capacity, and there were no capacity limits placed on most outdoor areas. To reiterate, we chose this period in order to understand potential relationships right at this crucial phase of the very first sign of drastic increases in cases in Harris County and across Texas.

**Figure 1:**
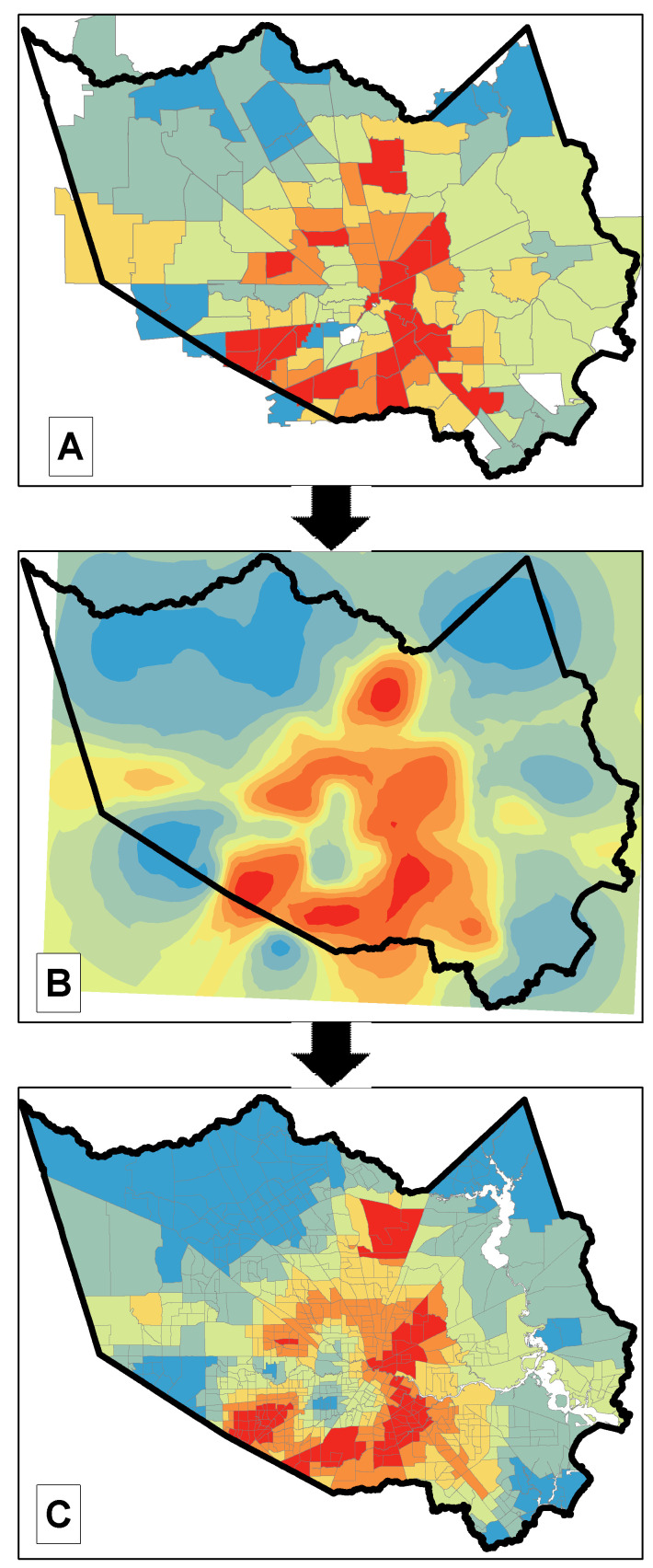
Predicting COVID-19 incidence at the census tract level. Steps involved in the disaggregation of zip-level COVID-19 community incidence data (observed data) to census tract estimates (predicted data) using the areal interpolation toolset in Esri’s ArcGIS Pro. (**A**) Observed data provided by the county: cases per 10,000 population at the zip code level. (**B**) Transform zip code level data to a prediction surface by using areal interpolation techniques. (**C**) Use the newly created prediction surface to estimate prevalence at the census tract level.

Our dependent variable was derived from COVID-19 cases reported for each zip code inside Harris County during our study period. To assemble the variable at the census tract level, we used the areal interpolation toolset in ArcGIS Pro 2.6 (Esri, Redlands, CA, USA) to disaggregate the provided zip-code level estimates down to census tracts units. The areal interpolation exercise involves a two-step process where, first, a prediction surface is created from the source geographic unit (here, the zip code), and second, the prediction surface is averaged within the target geographic unit (here, the census tract) [37]. See Figure 1.

We used the Geostatistical Wizard in ArcGIS Pro 2.6 (ESRI, Redlands, CA, USA) to implement the areal interpolation tool. We set the wizard to use the “event” input source data type, same data type recommended for overdispersed Poisson counts. The visual variography tools available in the Geostatistical Wizard are used to build a valid model in order to fit the data well and obtain accurate predictions. For our study, we used a K-Bessel model type and adjusted several variography parameters, including lattice spacing (x = 1500), lag size (x = 1800), and number of lags (x = 15). The cross-validation statistics are typically used to determine how well the interpolation models fit a dataset with an ideal standardized root mean square of 1.0. With our adjustments, the standardized root mean square for our interpolation model reached 0.944. Comprehensive discussions on areal interpolation, similar to our approach, have been previously presented [38,39,40].

### 2.3. Independent Variables

We assembled a total of 29 neighborhood-level characteristics under seven domains, including race/ethnicity and nativity (six variables), socioeconomic disadvantage (one variable), disaster vulnerabilities (four variables), over 65 years old (four variables), occupation (seven variables), access to technology (three variables), and senior care facilities (four variables). The specific measures that we examined under each domain, many being SDOH, are potentially relevant to our understanding of how neighborhood-level characteristics are associated with the community spread of COVID-19. These measures, shown in Table 1, were retrieved from the USA Census Bureau’s 2014–2018 American Community Survey (ACS) 5-year estimates dataset. The ACS is a nationwide survey that collects and produces information on social, economic, housing, and demographic characteristics about USA population every year. Over 3.5 million households across the USA participate in the ACS annually [41]. The ACS estimates are summarized to specific geographic levels, including the census tract.

All the neighborhood-level measures, except the area deprivation index (ADI), were used as retrieved from the ACS. The ADI is a composite measure of neighborhood socioeconomic disadvantage that relies on 17 Census measures (Supplementary File 1) from four major categories: poverty, housing, employment, and education [42,43]. We computed ADI for our analysis using the protocol developed and validated by Singh [43]. Specifically, our variable was calculated using 2014–2018 ACS dataset considering only census tracts within our study area (Harris County). We then grouped the outcome of the variable into deciles for ease of analysis.

### 2.4. Data Analysis

We relied on Poisson-based modeling protocols for all analysis steps given that our dependent variable is count-based data containing only non-negative integer values. Furthermore, to address the evidence of overdispersion observed in our dataset—the variance of the dependent variable is greater than the mean—we used the negative binomial regression (NBR) technique to analyze the variations in COVID-19 community incidence across Harris County census tracts, given specific independent explanatory neighborhood-level characteristics. The NBR is a regression modeling technique based on the Poisson-gamma mixture distribution, allowing the variance to have a much wider scope than is allowed by the Poisson distribution [44,45]. Technically, in the basic Poisson distribution, it is assumed that each count occurs over a small interval of time, area, or volume (TAV)—so small that the interval = 1. However, where unequal periods of TAV exist, an offset must be given in the model. Given unequal census tract populations in Harris County, the NBR model was applied to the count of COVID-19 cases in each census tract while population was used as an offset term (also called the “exposure variable”) [44].

To arrive at the final model, we followed a two-step approach. First, we assembled the variables under their respective domains and entered them into a multivariable model phase (domain-specific). We used the backward elimination process for domain-level variable selection. With backward elimination, variables were removed sequentially, starting with the highest *p*-value and continuing until only the statistically significant SDOH measures remained (passing *p*-value less than 0.05). After determining the model based on our selection, we inspected the results for multicollinearity. We removed any variable with a VIF ≥ 5.0 and re-ran the model. Second, the variables that passed the domain-specific multivariable selection phase were all entered into a single (domain-agnostic) multivariable regression phase— using backwards stepwise selection with the passing *p*-value < 0.05 and unacceptable VIF ≥ 5.0 here, too. All analyses were completed using Stata 16.0 (Stata Corp, College Station, TX, USA). We did not employ any technique for multiple comparisons because statistical tests were run within each domain separately, reducing our number of comparisons. Additionally, the two-step approach we used allowed us to select variables that were included in the final model, as opposed to just listing a bunch of variables.

In addition to the aspatial global Poisson regression analyses described above, we used the geographically weighted Poisson regression (GWPR) to identify spatial dependencies in the relationship between the neighborhood-level SDOH characteristics and COVID-19 community incidence. The GWPR technique extends the conventional regression framework by allowing local variations in rates of change among areas so that the coefficients in the model are specific to a location (i.e., local coefficients) rather than being global estimates [46,47]. These local beta (*β*) coefficients identify neighborhoods where the exposure–outcome relationships are strongest or weakest, or neighborhoods where relationships diverge from what was observed in global models. In essence, GWPR exposes spatial variations in the exposure–outcome relationship that global modeling techniques overlook [46,47]. Parameters of regression models for each regression point are estimated based on nearby observations, whereby data on closer census tracts have greater effect on estimates for any given tract than data for farther census tracts. Geographic weights are identified from a kernel function. The bi-square kernel uses an explicit threshold, assigning a weight of zero to any data observed outside of the bandwidth, and an adaptive kernel is appropriate when the regression points are irregularly positioned [18], as is the case in our study area. We used an adaptive bi-square kernel and the “golden section search” function in GWR4 in order to select an optimal number of *k* neighbors to be included in the local model fitting. The bandwidth selection protocol we used produced an optimal bandwidth of 54. In the case of the current analysis, only the variables that remained significant and non-collinear in the final stage of the aspatial global regression were allowed into the GWPR. Notably, although the variable “capacity of assisted living inside census tract” stayed significant in the final global regression model, it exhibited strong collinearity during the local GWPR regression run and was therefore excluded from the analysis. We ran the GWPR with the GWR4 software (Arizona State University, Phoenix, AR, USA) (http://geodacenter.asu.edu/gwr). Details about the GWR4 software settings have been previously described [48]. The local coefficients that resulted from the GWPR model using the GWR4 software were mapped in ArcGIS Pro (Esri, Redlands, CA, USA).

For all the Poisson regression analyses described above, effect estimates were expressed in terms of relative risk (incidence rate ratios = IRR) by exponentiating the Poisson regression coefficient. This was interpreted as an increase or a decrease in the risk of COVID-19 incidence associated with a 10-unit change in the independent variable.

## 3. Results

**Figure 2:**
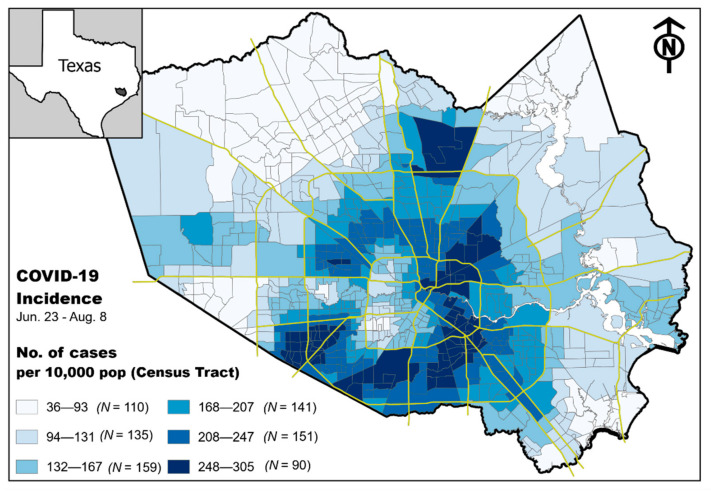
Maps of Texas showing Harris County and a zoomed-in version of the county. Choropleth map depicts the incidence of COVID-19 (cases per 10,000 population) between 23 June 2020 and 3 August 2020 at the census tract level (*N* = 786).

We observed a non-random pattern of incidence rate of COVID-19 at the census tract level in Harris County, Texas. Analysis of the incidence rate map suggest that higher rates of disease are more common in specific and highly focal areas located in the eastern–central portion of the county, as well as areas to the north and south (Figure 2).

### 3.1. Exploring Relationships between COVID-19 Incidence and Individual Neighborhood Factors

Between 23 June 2020 and 3 August 2020, 70,396 incident cases of COVID-19 were reported in Harris County. We conducted an initial univariate analysis to identify the neighborhood variables that were associated with COVID-19 incidence at the census tract level. Out of the 29 variables examined, only 3 were not associated with the outcome, including: the percentage in health care occupation, number of nursing homes, and capacity of nursing homes inside the census tract. The remaining 26 variables had a mixture of positive and negative relationships with COVID-19 community incidence (Table 2). 

### 3.2. Domain-Specific Relationships between Neighborhood Factors and COVID-19

We then conducted backwards stepwise negative binomial regression analysis with only the variables in their respective domains. These subsequent models resulted in the removal of an additional 11 variables. Of the removed variables, 10 were excluded based on *p*-value criteria and one was excluded due to collinearity. In the race/ethnicity and nativity domain, the percentage of the Asian population, percentage of the non-Hispanic white population, and percentage of other race (or two or more races) population were removed from the analysis. From the occupation domain, the percentage in healthcare, human services, and food preparation were removed from the analysis. In the access to technology domain, the percentage of households that have no computer, smartphone, or tablet was removed from the analysis. In the over 65 years old domain, percent of the population over 65 living in quarters was removed. In the senior care facilities domain, the number of assisted living facilities, number of nursing homes, and capacity of nursing homes in a census tract was removed. All variables in the disaster vulnerabilities and socioeconomic disadvantage remained in the analysis (Table 3). 

### 3.3. Across-Domains Relationships between Neighborhood Factor and COVID-19

Our final model was built considering the 18 remaining variables after univariate and domain-specific model selection. Backwards stepwise model selection and assessment for multicollinearity removed an additional 10 and one independent variables, respectively. Our final model contains the percentage of the Black or African American population, the percentage of the foreign-born population, ADI, the percentage of households with no vehicle available, the percentage of the population over 65 years old, the percentage of education/training/library occupation, and capacity of assisted living inside census tract (Table 4).

**Figure 3:**
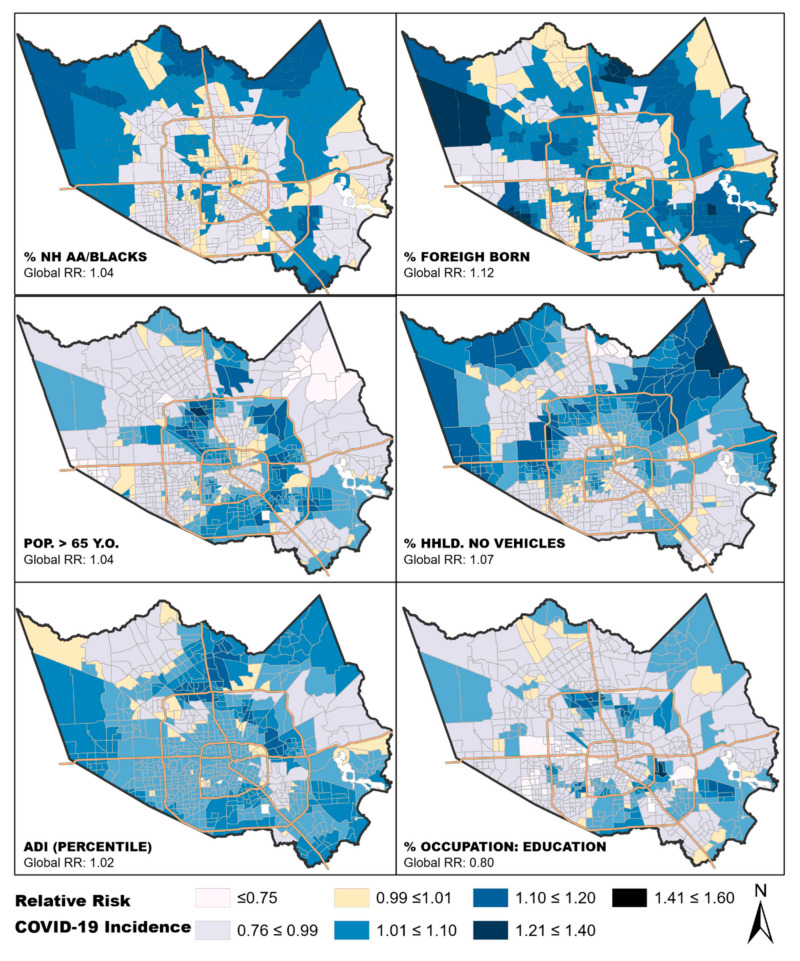
Maps of Harris County census tracts that show local associations between the variables in the final aspatial model and COVID-19 incidence. The local beta coefficients were exponentiated (RR) to show the sensitivity of COVID-19 incidence to a change of 10-unit difference in each of the neighborhood characteristics shown above, specific to each census tract. The middle class (0.99–1.01) crosses 1.0. To simplify interpretation, the same legend was applied to all maps. Values less than 0.99 suggest census tracts where increase in the proportion of the independent variable is associated with decreased RR for COVID-19, while values above 1.01 suggest increased RR for COVID-19 with increased proportion of the independent variable (10-unit increase).

In the GWPR, we tested for local associations between the variables in our final run of the global regression model, except for “capacity of assisted living inside census tract”. We observed that the relationship between each variable and COVID-19 incidence was spatially dynamic. In all cases, the coefficients varied across Harris County census tracts and ranged from decreased risk in some tracts to increased risk in others (Table 5). The spatial patterns of the local GWPR regression outcomes were displayed using a choropleth map (Figure 3). The Akaike Information Criterion (AIC) goodness-of-fit and AICc indicators were compared between the local and global models, and the GWPR model had a significantly smaller AICc and AIC (Table 6). This suggests that the GWPR model fit the data better, i.e., had better explanatory power.

## 4. Discussion

Central to effective control measures for a pandemic is understanding the epidemiology of transmission in the community. Our study joins the list of recent and growing research studies examining various aspects of the relationships between socioeconomic/environmental factors and the incidence of COVID-19 [11,49]. We used a series of aspatial and spatially weighted regression models to identify neighborhood-level characteristics that are associated with higher COVID-19 incidence at the census tract level. Our study area, Harris County, is a major USA metropolitan county. Across our several analysis steps, characteristics that represent either minority population or socioeconomic disadvantage had positive associations with COVID-19 incidence. Out of 29 variables that we considered in the analysis, 7 remained significant correlates of COVID-19 community incidence in our final global model: the percentage of the Black or African American population, the percentage of the foreign-born population, ADI, the percentage of households with no vehicle available, and the percentage of the population over 65. Two variables found to be protective were the percentage in education, training, or library occupation and capacity of assisted living. By understanding variables that correlate with community transmission, we can better direct resources, expand testing capacity, and focus disease control measures.

Conducting this analysis at the neighborhood level is a critical component of this study. Over the last decade, scholars have argued for and validated the importance of examining the impact of “place-based” socioenvironmental factors on health outcomes [50,51]. In this regard, the places where people live, work, and play are frequently considered, though the residential neighborhood are appropriately the typical unit of analysis. Neighborhoods are not randomly constructed; they are patterned around social status, ethnicity, and income [52]. These factors strongly influence an individual’s determinants of health and have been shown to correlate with health status and overall mortality rates [53]. Understanding how neighborhood factors influencing transmission of this novel disease will be critical in preventing future outbreaks.

One variable that was highlighted in our analysis and showed the strongest relationship to increased risk of COVID-19 was the area deprivation index (ADI). This index is a validated composite measure of neighborhood socioeconomic inequalities and disadvantage [42]. Significant inequalities have been found to influence historic pandemics. Sydenstricker, as far back as 1931, demonstrated inequalities in the working-class population of the USA during the Spanish influenza pandemic of 1918–1919 [54]. Contemporary evidence has also shown that these inequalities during times of pandemics existed in terms of key spatial attributes such as affluence of neighborhoods, socioeconomic strata, and the urban–rural gradient [55,56,57,58]. The ADI has been used to examine disease risk factors [59], predict healthcare utilization [60], and understand healthcare disparities [43,61]. Recently, Singh and colleagues recognized the ADI for having been a powerful tool for documenting and monitoring population health inequalities across time and space in the USA [61]. ADI was one of the strongest correlates of high COVID-19 incidence at the community level in our analysis. Community-level poverty can influence health on many levels. It affects everything from health care utilization, access to healthy foods, recreational activities, built environment, and neighborhood safety. This index likely represents a very complex relationship between community and health. Our findings are consistent with those of several recent studies that have indicated that factors associated with social and economic disadvantage have been associated with COVID-19 [19,62].

Our analysis also indicated that racial/ethnic composition and nativity of neighborhood populations were significantly correlated with COVID-19 incidence. The underlying causes of health disparities among racial minorities in the US are likely complex and cannot be easily summarized. They derive from relationships among social structure, cultural norms, racism, and socioeconomic factors. This is likely why the variable representing the percentage of the Black or African American population and the percentage of the foreign-born population remained significant in our analysis, but many other factors that contribute to inequality were found to be not significant in the final model. Further research to understand these community-level drivers of health inequality is critical to determine points of intervention to prevent disease transmission and reduce the disproportionate morbidity and mortality that these communities have experienced as a result of COVID-19.

These findings have potential relevance to the release of COVID-19 vaccines as part of Operation Warp Speed. Given the potential COVID-19 risks to African American and foreign-born populations, prioritizing vaccine access in Houston and Harris County to such vulnerable groups presents special urgencies. Of particular concern are recent reports of COVID-19 vaccine hesitancy in African American populations [63]. Still another issue are the high rates of COVID-19 deaths among both African American and Hispanic groups at younger ages (<65 years old) compared to the non-Hispanic Whites, such that relying on 65-year age cut-offs for vaccinations might miss highly vulnerable subpopulations [36].

Our geographically weighted Poisson regression analysis produced local beta coefficients for each of the census tracts in our study area. This tool allows for visualization of the impact of each independent variable in individual neighborhoods. Interestingly, the impact of ADI appears to be homogeneous across our study area, indicating that variation in ADI affects neighborhoods equally regardless of other factors. It appears that an increased percentage of African American or foreign-born population within the community has a greater impact in the less densely populated periphery of the county. Conversely, neighborhoods with larger populations of residents over 65 years old had a greater impact in parts of the county that are more densely populated. Conducting this local analysis by census tract provides a visual output of each variable’s impact that is easily interpreted for directing public health interventions.

Our study has some noteworthy limitations. Our dependent variable, COVID-19 incidence, was derived from publicly available data provided by public health authorities in Harris County and the City of Houston. While this is currently the only source of COVID-19 incidence data, we cannot ensure that it is always timely and accurate. Inadequate access to testing, delayed testing results, and backlogged data could affect our data quality. The independent variables considered in this analysis may not represent a comprehensive list of all factors influencing COVID-19 transmission in this community. While we believe that we accurately represented likely risk factors, we cannot rule out other influences. As with any epidemiologic analysis using non-individual level estimates, our analysis is susceptible to ecologic fallacy. Additionally, this is a correlational study, and therefore, causal inference cannot be made; as such, coefficients should be cautiously interpreted. We believe that novel analytic workflow and the importance of the findings of this analysis for local public health officials outweighs these limitations. While these limitations are important to consider, we also recognize that the large sample size of cases strengthens the power of our study.

The assessment of disparities in health outcomes requires the ability to understand spatial and spatially driven structures that influence the exposure–disease relationships. Understanding health disparities through spatial processes is perhaps especially useful in societies where heterogeneous neighborhoods composed of diverse groups are seldom the norm. Of course, the utility of geospatial analytics and processes in this regard should not just be for the sake of itself. Findings from this type of geographically weighted analyses should provide insight into neighborhood-level drivers of infection that would have otherwise been missed by public health officials. This powerful analytic process can provide information to effectuate holistic policy prescriptions that are often operationalized in geographical space [64]. The utility of spatial analyses for understanding and managing the COVID-19 pandemic may create levels of structural resources that, when adequately leveraged, could facilitate effective intervention strategies, allocation of resources, and delivery of care to all, and especially those disproportionately burdened by the pandemic.

## 5. Conclusions

In conclusion, we believe that our study provides evidence that geospatial analysis can be a powerful tool for determining neighborhood-level correlates of COVID-19. During a global pandemic of a novel virus, both resources and knowledge about viral transmission dynamics are limited. This type of analysis could provide real-time information to allow for data-driven decision making by local public health officials. We believe this analysis provides critical information for controlling the current pandemic and serves as a proof of concept for use in future disaster response scenarios.

## Figures and Tables

**Table 1:** List of Independent Variables.

**Race/Ethnicity and Nativity**
% Non-Hispanic white pop.
% Black or African American pop.
% Asian pop.
% Other race + two or more races pop.
% Hispanic or Latino pop.
% Foreign-born pop. who is not a United States citizen
**Socioeconomic Disadvantage**
Area Deprivation Index (ADI) *
**Disaster Vulnerabilities**
% of households with no vehicle available
% of adults 18 y and over who have limited English ability
% of pop. with a disability
% of pop. with no health insurance coverage
**Over 65 Years Old**
% of pop. that is 65 y and over
% of pop. 65 y and over who live alone
% of pop. 65 y and over with a disability
% of pop. 65 y and over living in quarters
**Occupation**
% Health and healthcare support
% Human Services
% Management, science, technology
% Mobile workers, construction, maintenance
% Food preparation
% Personal care
% Education/Training/Library
**Senior Care Facilities**
No. of assisted living inside census tract
Capacity of assisted living inside census tract
No. of nursing homes inside census tract
Capacity of nursing homes inside census tract
**Access to Technology**
% of Households that have no computer, smartphone, or tablet
% Households with cellular data plan with no other type of internet subscription
% of Households with no internet access

* ADI is a composite indicator of socioeconomic disadvantage that is based on 17 census indicators from four major categories: poverty, housing, employment, and education. See Supplementary File 1 for more details.

**Table 2:** Univariate model of neighborhood factors and the community incidence of COVID-19 in Harris County, Texas, between 23 June 2020 and 3 August 2020. Harris County census tracts (*N* = 786).

Independent Variables	Coeff.	Coeff. 95% CI		IRR	IRR 95% CI		*p*-Value
Race, ethnicity, nativity							
% Non-Hispanic White pop	−0.099	−0.108	−0.091	0.906	0.989	0.991	<0.001
% Black or African American pop	0.043	0.029	0.056	1.044	1.003	1.006	<0.001
% Asian pop.	−0.105	−0.139	−0.071	0.900	0.986	0.993	<0.001
% Other race + two or more races pop	−0.755	−0.906	−0.605	0.470	0.913	0.941	<0.001
% Hispanic or Latino pop	0.074	0.064	0.083	1.077	1.006	1.008	<0.001
% Foreign-born pop. who is not a United States citizen	0.109	0.090	0.128	1.115	1.009	1.013	<0.001
Socioeconomic disadvantage							
Area Deprivation Index (ADI)	0.710	0.627	0.792	2.034	1.873	2.208	<0.001
Disaster vulnerabilities							
% of households with no vehicle available	0.266	0.225	0.307	1.305	1.023	1.031	<0.001
% of adults 18 y and over who have limited English ability	0.104	0.090	0.118	1.109	1.009	1.012	<0.001
% of pop. with a disability	0.153	0.091	0.215	1.166	1.009	1.022	<0.001
% of pop. with no health insurance coverage	0.186	0.166	0.206	1.204	1.017	1.021	<0.001
Over 65 years old							
% of pop. that is 65 y and over	−0.097	−0.150	−0.043	0.908	0.985	0.996	<0.001
% of pop. 65 y and over who lives alone	0.033	0.013	0.052	1.033	1.001	1.005	0.001
% of pop. 65 y and over with a disability	0.063	0.044	0.083	1.065	1.004	1.008	<0.001
% of pop. 65 y and over living in quarters	0.035	−0.005	0.075	1.035	0.999	1.007	0.090
Occupation							
% Health and healthcare support	−0.025	−0.148	0.098	0.976	0.985	1.010	0.693
% Human services	−0.109	−0.173	−0.045	0.897	0.983	0.996	0.001
% Management, science, technology	−0.075	−0.084	−0.066	0.928	0.992	0.993	<0.001
% Mobile workers, construction, maintenance	0.034	0.016	0.052	1.034	1.002	1.005	<0.001
% Food preparation	0.260	0.194	0.326	1.296	1.020	1.033	<0.001
% Personal care	0.215	0.107	0.323	1.240	1.011	1.033	<0.001
% Education/Training/Library	−0.427	−0.477	−0.377	0.652	0.953	0.963	<0.001
Access to technology							
% of households that have no computer, smartphone, or tablet	0.196	0.172	0.220	1.217	1.017	1.022	<0.001
% Households with cellular data plan; no other type of internet	0.182	0.159	0.205	1.200	1.016	1.021	<0.001
% of households with no internet access	0.161	0.144	0.179	1.175	1.014	1.018	<0.001
Senior care facilities							
No. of assisted living inside census tract	−0.314	−0.619	−0.009	0.731	0.940	0.999	0.044
Capacity of assisted living inside census tract	−0.016	−0.023	−0.010	0.984	0.998	0.999	<0.001
No. of nursing homes inside census tract	−0.402	−1.084	0.280	0.669	0.897	1.028	0.248
Capacity of nursing homes inside census tract	−0.002	−0.007	0.004	0.998	0.999	1.000	0.575

Note: The relative risk for COVID-19 is reported per 10-unit increase in the magnitude of each neighborhood-level explanatory variable. CI, Confidence Interval; IRR, Incidence Rate Ratios.

**Table 3:** Domain-specific multivariable relationships between neighborhood factors and the community incidence of COVID-19 in Harris County, Texas, between 23 June 2020 and 3 August 2020. Harris County census tracts (*N* = 786).

Independent Variable	Coeff.	Coeff. 95% CI		IRR	IRR 95% CI		*p*-Value
Race, ethnicity, nativity							
% Non-Hispanic white pop.	n.s.						
% Black or African American pop.	0.0831	0.072	0.094	1.087	1.075	1.098	<0.001
% Other race + two or more races pop.	n.s.						
% Hispanic or Latino pop.	0.0739	0.064	0.084	1.077	1.066	1.088	<0.001
% Foreign-born pop. not a United States citizen	0.0635	0.044	0.083	1.066	1.045	1.087	<0.001
Socioeconomic disadvantage							
Area Deprivation Index (ADI)	0.7100	0.630	0.790	2.034	1.878	2.203	<0.001
Disaster vulnerabilities							
% of households with no vehicle available	0.1268	0.086	0.168	1.135	1.090	1.182	<0.001
% of adults 18 y and over who have limited English ability	0.0324	0.008	0.057	1.033	1.008	1.058	0.009
% of pop. with a Disability	0.1125	0.054	0.171	1.119	1.056	1.186	<0.001
% of pop. with No health insurance coverage	0.1166	0.079	0.154	1.124	1.082	1.167	<0.001
Over 65 years old							
% of pop. that is 65 y and over	−0.0949	−0.111	−0.079	0.910	0.895	0.924	<0.001
% of pop. 65 y and over who lives alone	0.0306	0.025	0.036	1.031	1.026	1.036	0.017
% of pop. 65 y and over with a disability	0.0585	0.053	0.064	1.060	1.054	1.066	<0.001
% of pop. 65 y and over living in quarters	n.s.						
Occupation							
% Healthcare	n.s.						
% Human services	n.s.						
% Management, science, technology	−0.0442	−0.060	−0.029	0.957	0.942	0.972	<0.001
% Mobile workers, construction, maintenance	0.0580	0.043	0.073	1.060	1.044	1.075	<0.001
% Food preparation	n.s.						
% Personal care	0.0996	0.008	0.191	1.105	1.008	1.210	<0.001
% Education/Training/Library	−0.2612	−0.345	−0.177	0.770	0.708	0.838	<0.001
Access to technology							
% of households that have no computer, smartphone, or tablet	n.s.						
% Households with cellular data plan; no other type of internet	0.0943	0.068	0.121	1.099	1.070	1.128	<0.001
% of Households with no internet access	0.1144	0.093	0.136	1.121	1.098	1.145	<0.001
Senior care facilities							
No. of assisted living inside census tract	n.s.						
Capacity of assisted living inside census tract	−0.016	−0.023	−0.010	0.984	0.978	0.990	<0.001
No. of nursing homes inside census tract	n.s.						
Capacity of nursing homes inside census tract	n.s.						

Note: The relative risk for COVID-19 is reported per 10-unit increase in the magnitude of each neighborhood-level explanatory variable, holding all other variables in the model constant. CI, Confidence Interval; IRR, Incidence Rate Ratios; n.s., not significant.

**Table 4:** Across-domains multivariable relationships between neighborhood characteristics and the community incidence of COVID-19 in Harris County, Texas, between 23 June 2020 and 3 August 2020. Harris County census tracts (*N* = 786).

Independent Variable	Coeff.	Coeff. 95% CI		IRR	IRR 95% CI		*p*-Value
% Black or African American pop.	0.0267	0.0131	0.040	1.027	1.013	1.041	<0.001
% Foreign-born pop. not a United States citizen	0.1066	0.0806	0.133	1.112	1.084	1.142	<0.001
Area Deprivation Index (ADI)	0.2709	0.1700	0.372	1.311	1.185	1.450	<0.001
% of households with no vehicle available	0.0741	0.0327	0.116	1.077	1.033	1.122	<0.001
% of pop. that is 65 y and over	0.0588	0.0085	0.109	1.061	1.009	1.115	0.022
% Education/Training/Library occupation	−0.1941	−0.2497	−0.139	0.824	0.779	0.871	<0.001
Capacity of assisted living inside census tract	−0.0077	−0.0130	−0.002	0.992	0.987	0.998	0.004

Note: The relative risk for COVID-19 is reported per 10-unit increase in the magnitude of each neighborhood-level explanatory variable, holding all other variables in the model constant. CI, Confidence Interval; IRR, Incidence Rate Ratios.

**Table 5:** Geographically weighted Poisson regression (GWPR) modeling summary statistics.

Local Terms	Mean	STD	Min.	Lower Quartile	Median	Upper Quartile	Max.
Intercept	−47.19	8.07	−81.00	−51.51	−45.18	−41.65	−22.34
% NH Black or African American pop.	0.00	0.06	−0.24	−0.03	−0.01	0.03	0.18
% Foreign-born pop. not a United States citizen	0.02	0.08	−0.29	−0.02	0.02	0.07	0.29
Area Deprivation Index (ADI)	0.06	0.07	−0.18	0.02	0.05	0.10	0.34
% of households with no vehicle available	0.01	0.12	−0.48	−0.05	0.01	0.07	0.38
% of pop. that is 65 y and over	−0.01	0.13	−0.43	−0.09	−0.01	0.07	0.34
% Education/Training/Library occupation	−0.06	0.14	−0.65	−0.12	−0.05	0.02	0.47

**Table 6:** Comparison between GWPR and global Poisson regression modeling results.

Indicators	GWPR	Global Model
AIC	1422.19	6242.63
AICc	1254.09	6242.49

## Data Availability

The data presented in this study are available on request from the corresponding author.

## Supplementary Materials

The following are available online at https://www.mdpi.com/1660-4601/18/4/1495/s1, Table S1: Variables used for the ADI. A total of 17 census variables drawn from 4 major categories, including poverty, housing, employment, and education.
