# Supplementary material for: COVID-19 Community Incidence and Associated Neighborhood-Level Characteristics in Houston, Texas, USA
Source: Int J Environ Res Public Health. 2021 Feb 4;18(4):1495. doi: 10.3390/ijerph18041495 (PMC7915818; doi:10.3390/ijerph18041495)
Supplement: Supplementary file 1 [file ijerph-18-01495-s001.pdf]

International Journal of
Environmental Research and Public Health

MDPI

Supplementary Table S1. Variables used for the ADI. A total 17 census variables drawn from four major categories, including; poverty, housing, employment, and education.

**Poverty**	
1	Median family income, $
2	Income disparity
3	Families below poverty level
4	% population below 150% poverty threshold
5	% Single parent households with dependents <18
6	% Households without a motor vehicle
7	% Households without a telephone
8	% Occupied housing units without complete plumbing
**Housing**	
9	% Owner occupied housing units
10	% Households with >1 person per room
11	Median monthly mortgage, $
12	Median gross rent, $
13	Median home value, $
**Employment**	
14	% Employed person 16+ in white collar occupation
15	% Civilian labor force unemployed (age 16+)
**Education**	
16	% Population aged 25+ with <9year education
17	% Population aged 25+ with at least a high school education
